# How can stroke patients have better accessibility to stroke fast track system in Thailand?

**DOI:** 10.1186/1471-2458-14-S1-P4

**Published:** 2014-01-29

**Authors:** Jiraluck Nontarak, Sirinad Nipaporn, Samrit Srithamrongsawat, Vinai Leesmidt, Supasit Pannarunothai

**Affiliations:** 1 Faculty of Medicine, Naresuan University, Phitsanulok 65000, Thailand; 2 Health Insurance System Research Office, Nonthaburi 11000, Thailand; 3 Klong Klung Hospital, Kamphaeng Phet, Thailand

## 

In Thailand, 2005, the mortality rate of stroke was 24.3 per 100,000 populations and since then it has continuously increased. The prevalence of stroke increased from 216.6 to 307.9 in 2008 to 2010. Diabetes, hypertension and hyperlipidaemia are the root cause of stroke and myocardial infarction. To prevent stroke is to prevent complication, and in order to do so, it needs the implementation of effective stroke fast track policy. In 2009, the National Health Security Office (NHSO) launched stroke fast track policy by supporting the costs of thrombolytic agent (rt-PA), computed tomography scan, physical therapy and home visit. The policy aimed to reduce mortality rate and disability for patients covered by universal coverage scheme (UCS). However, this special management for stroke fast track are not covered by the civil servant medical benefit scheme (CSMBS) and social security office (SSO).

Up till now, the utilization rate of thrombolytic agent is still low. One factor is delayed hospital presentation in patients with such condition. This is because of lack of knowledge and understanding of early signs and symptoms of stroke. However, stroke fast track is the best practice to increase the accessibility and reduce the severity and disability of patients.

The question remains whether stroke fast track policy is successful, and whether it can improve the accessibility to care. Which factors influence policy implementation? It is therefore important to study the process of this policy in order to develop policy implementation.

